# Past-month binge drinking and cannabis use among middle-aged and older adults in the United States, 2015–2019

**DOI:** 10.1016/j.alcohol.2022.07.006

**Published:** 2022-08-05

**Authors:** Wayne E. Kepner, Benjamin H. Han, Daniel Nguyen, Stacy S. Han, Francisco A. Lopez, Joseph J. Palamar

**Affiliations:** a University of California San Diego, Department of Medicine, Division of Geriatrics, Gerontology, and Palliative Care, 9500 Gilman Drive, San Diego, CA 92093, United States; b New York University Grossman School of Medicine, Department of Population Health, 180 Madison Avenue, New York, NY 10016, United States

**Keywords:** aging, binge drinking, cannabis, chronic disease

## Abstract

The aging United States population, which includes the large Baby Boomer generation, is leading to an increasing number of middle-aged and older adults who engage in psychoactive substance use. Due to the size of this cohort, and their changing attitudes around psychoactive substance use, there has been a sharp increase in prevalence of past-month cannabis use among adults aged ≥50; however, little is understood about recent trends in the use of both cannabis and excess alcohol use, such as binge drinking, in this population. The current use of both alcohol and cannabis has important health implications for older adults, given their higher prevalence of chronic diseases and prescribed medications. This study aimed to 1) estimate national trends among older adults who report both past-month binge drinking and cannabis use, and 2) examine correlates of reporting both. We examined aggregated data from a nationally representative sample of noninstitutionalized adults aged ≥50 from the 2015 to 2019 National Survey on Drug Use and Health. During the study period, there was an estimated 2.5% increase (a 64.1% relative increase) in past-month cannabis use (linear trend *p* < 0.001), a non-significant decrease in past-month binge drinking, and a 0.5% estimated increase in both past-month cannabis use and binge drinking (a 26.3% relative increase) (*p* = 0.03). The sharpest increase in both past-month cannabis use and binge drinking was among adults aged ≥65, with the estimated prevalence increasing from 0.2% in 2015 to 1.1% in 2019 (a 450% relative increase, *p* < 0.001). Those engaging in past-month binge drinking and cannabis use were more likely to be younger, male, non-Hispanic Black, use tobacco, and report past-year mental health treatment. Results suggest that the prevalence of both past-month cannabis use and binge drinking among middle-aged and older adults increased between 2015 and 2019, especially among adults aged ≥65, which indicates an increased need to screen for both excess alcohol and cannabis use to minimize potential harm.

## Introduction

Over the past decade, the proportion of older adults who use cannabis or alcohol has risen sharply (Grant et al., 2017; Han, Moore, Sherman, Keyes, & Palamar, 2017; Han, Sherman, et al., 2017). Past-year cannabis use among adults aged ≥65 in the United States (US) rose from 0.4% in 2006 to 4.2% in 2018 (Han, Moore, et al., 2017; Han & Palamar, 2020), and past-month binge drinking increased from 8.1% in 2005/2006 to 11.1% in 2019 (Al-Rousan, Moore, Han, & Palamar, 2022; Han, Moore, et al., 2017). With the increase in both cannabis and alcohol use, associations have been established between cannabis use and binge drinking among older adults (Al-Rousan, Moore, Han, Ko, & Palamar, 2022; Han, Moore, et al., 2017; Han, Moore, Ferris, & Palamar, 2019; Han, Sherman, et al., 2017). However, a more comprehensive examination of the current use of both substances by older adults is needed. The relationship between cannabis and alcohol use is complex and the use of both cannabis and alcohol can be more problematic than singular use of either substance (Gunn, Aston, & Metrik, 2022). Generally, concurrent use is defined as the use of alcohol and cannabis in the same time period (with periods varying depending on the study), while simultaneous use is use at the “same time.” One study demonstrated that respondents who consumed five or more drinks in a day were more likely to use cannabis and alcohol concurrently and that such use was associated with depression (Midanik, Tam, & Weisner, 2007). Additionally, individuals with current or lifetime diagnoses of cannabis use disorder are at an increased likelihood of having an alcohol use disorder (Agosti, Nunes, & Levin, 2002; Stinson, Ruan, Pickering, & Grant, 2006). Concurrent use of cannabis and alcohol may also increase risk for unsafe driving (Terry-McElrath, O'Malley, & Johnston, 2014). These studies suggest that the use of both alcohol and cannabis may have higher risks of adverse effects than the singular use of either substance, even if not used simultaneously (Yurasek, Aston, & Metrik, 2017).

While a cross-sectional study demonstrated that adults aged ≥35 who use cannabis were 3.7 times more likely to binge drink than those who did not report cannabis use (Vijapur, Levy, & Martins, 2021), other studies have shown that cannabis initiation is associated with a reduction in alcohol use in some populations. For example, a large Canadian study found that alcohol frequency and the number of drinks consumed decreased after medical cannabis initiation (Lucas, Boyd, Milloy, & Walsh, 2020). Approximately 29% fewer drinks were consumed on days cannabis was used. Similarly, participants were 2.1 times less likely to report engaging in binge drinking episodes on days they also used cannabis (Karoly, Ross, Prince, Zabelski, & Hutchison, 2021). These data suggest that individuals who use cannabis may experience a decrease in risky drinking behavior. Notably, studies on cannabis initiation and associations with risky drinking behavior among older adults are lacking.

Psychoactive substance use can have stronger effects on older adults since they are susceptible to age-associated physiological changes, and adverse effects from its use are particularly concerning for older adults living with chronic diseases (Kuerbis, 2020). For example, binge drinking may be particularly dangerous for older adults due to an age-related increase in brain sensitivity to alcohol (Van Skike & Matthews, 2021), and the addition of another psychoactive substance such as cannabis may add additional health risks (Han & Moore, 2018; Subbaraman & Kerr, 2015). While an abundance of research has focused on the effects of cannabis and alcohol in the general population, there may be distinct risks associated with such use by older adults (e.g., cognitive decline) that are not well understood. Further studies are needed to understand the potential risks of alcohol and cannabis use in the older adult population and any changing in patterns of use of both.

Limited research suggests that older adults are engaging in concurrent alcohol and cannabis use at increasing rates. Previously, one study found that among adults aged ≥50 participating in the 2005–2010 National Alcohol Survey in the US, an estimated 1.8% engaged in the use of cannabis and alcohol separately, while 2.4% used cannabis and alcohol at the same time in the past year (Subbaraman & Kerr, 2015). A more recent study that focused on a general population survey in Washington state found that among older adults who use cannabis, an estimated 50.8% of those aged 50–64 and 41.5% of those aged ≥65, “usually” or “always” co-use cannabis with alcohol. Furthermore, they found that among men aged ≥65, frequent cannabis use was positively associated with the use of alcohol (Subbaraman & Kerr, 2021). While older adults have a lower total estimated prevalence of concurrent cannabis and alcohol than young adults or middle-aged adults, recent data suggest that this behavior may be increasing.

Understanding the sociodemographic characteristics and recent trends in the current prevalence of both alcohol and cannabis use among older adults is essential for targeted public health and clinical interventions. Our main aim was to estimate national trends and characterize the sociodemographic characteristics of older adults who engage in both current (past 30-day) binge drinking and cannabis use. A secondary aim of this study was to examine the demographic correlates of people who report both past-month binge drinking and cannabis use. As such, we examined repeated cross-sectional data from a nationally representative sample of noninstitutionalized older individuals in the US aged ≥50.

## Materials and methods

### Data source

We examined aggregated data from respondents aged ≥50 from the 2015 to 2019 National Survey on Drug Use and Health (NSDUH) (Center for Behavioral Health Statistics and Quality; Substance Abuse and Mental Health Services Administration (SAMHSA), 2020). NSDUH is a repeated cross-sectional survey of noninstitutionalized individuals in the 50 US states and the District of Columbia. NSDUH obtains a nationally representative probability sample of individuals obtained through four stages. Surveys are administered via computer-assisted interviewing (conducted by an interviewer) and audio computer-assisted self-interviewing (ACASI). Sample weights are provided by NSDUH to address unit- and individual-level nonresponse.

### Measures

Participants are asked whether they have used alcohol and whether they have engaged in binge drinking in the past month. NSDUH defines past-month binge alcohol use based on the National Institute on Alcohol Abuse and Alcoholism's (NIAAA's) definition of consuming five or more alcoholic beverages on the same occasion for men and four or more on the same occasion for women (National Institute on Alcohol Abuse and Alcoholism [NIAAA], (2022)). Cannabis use is ascertained by asking about marijuana, hashish, pot, grass, and hash oil use either smoked or ingested in the past month. Past 30-day use was considered “current” use in this analysis as this definition is commonly used by NSDUH (Substance Abuse and Mental Health Services Administration, 2021).

Other covariates included in this analysis were sociodemographic characteristics including age range (pre-defined by NSDUH as 50 to 64 and ≥ 65), sex, race/ethnicity, and annual family income. Race/ethnicity was also dichotomized into non-Hispanic white vs. non-white for trend analysis, as some years had too few participants per cell to achieve reliable estimates. We also considered all-cause emergency department (ED) use in the past year and whether participants received any mental health treatment in the past year. Participants were also asked about past-month tobacco use. Finally, participants were asked whether they had ever been informed by a physician or other medical professional that they ever had the following medical diseases: asthma, cancer, cardiovascular disease, chronic obstructive pulmonary disease, cirrhosis, diabetes, hepatitis B or C, HIV/AIDS, hypertension, and kidney disease. To examine medical multimorbidity, we further recoded these variables to indicate whether respondents have been diagnosed with two or more chronic conditions.

### Statistical analysis

We estimated the prevalence of past-month cannabis use, binge drinking, and both cannabis and binge drinking (based on affirmative responses to both) across survey years. We then estimated prevalence of adults who engaged in both past-month binge drinking and cannabis use stratified by each level of sex, race/ethnicity, family income, chronic disease, tobacco use, mental health treatment, and all-cause ED use. We also calculated the absolute and relative change in prevalence between 2015 and 2019. Using logistic regression, we estimated whether there was a linear association between both cannabis use and binge drinking with time (year).

We then characterized differences in the above covariates between those who engaged in both past-month cannabis use and past-month binge drinking and compared them to those who did not report both. Comparisons were made using Rao Scott χ^2^ tests and multivariable generalized linear models using Poisson and log link to examine associations between independent variables (controlling for survey year) and reporting both past-month cannabis use and binge drinking among adults, resulting in adjusted prevalence ratios (aPRs) for each covariate. We used weights to account for the complex survey design, selection probability, non-response, and population distribution. This secondary analysis was exempt from review by the New York University Langone Medical Center institutional review board.

## Results

**Fig. 1.**
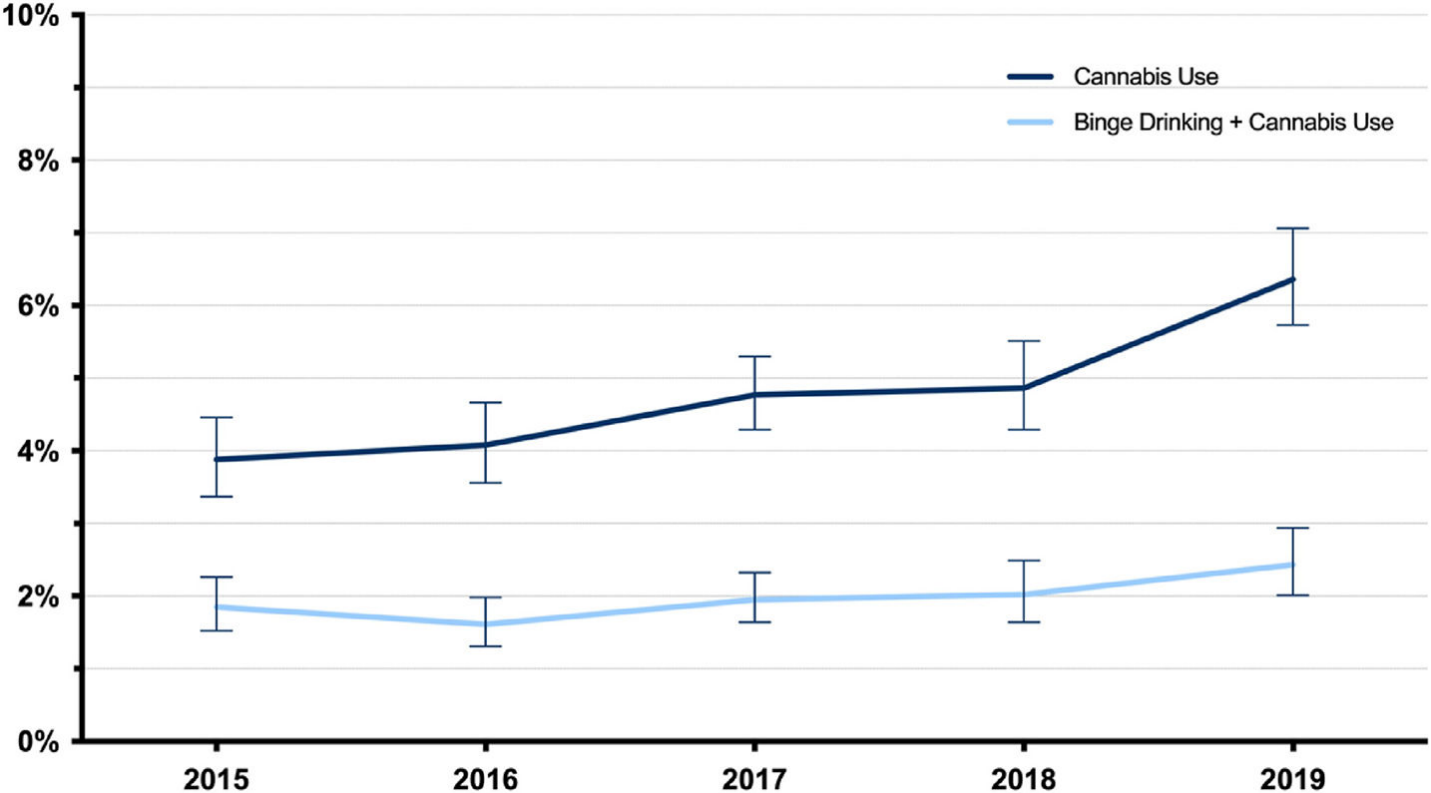
Trends in past-month cannabis use and both past-month cannabis use and binge drinking among adults aged ≥50, United States, 2015–2019.

Based on data from the 44,007 respondents, the aggregated prevalence of past-month binge drinking was 17.5%; an estimated 4.8% engaged in past-month cannabis use, and an estimated 2.0% engaged in both. The estimated prevalence of past-month cannabis use increased from 3.9% in 2015 to 6.4% in 2019 (*p* < 0.001), an increase of 64.1% (Fig. 1). The estimated prevalence of past-month binge drinking decreased from 18.1% in 2015 to 17.3% in 2019 (*p* = 0.46), a relative decrease of 4.4%. The estimated prevalence of both past-month binge drinking and cannabis use, however, significantly increased from 1.9% in 2015 to 2.4% in 2019 (*p* = 0.03), a relative increase of 26.3% (Fig. 1).

Table 1 presents trends of subgroups of adults aged ≥50 who reported both past-month binge drinking and cannabis use. There were significant increases among adults in both age groups examined. Specifically, there was a large increase detected among those aged ≥65 from 0.2% in 2015 to 1.1% in 2019 (a 450.0% relative increase; *p* < 0.001). Concurrent use increased among those identifying as non-Hispanic white (a 33.3% increase; *p* = 0.03) and among those with a family income <$20,000 (an 86.4% increase; *p* = 0.01). In addition, concurrent use increased among adults who have visited an ED in the past year (a 45.0% increase; *p* = 0.03).

Table 2 presents correlates of engaging in both past-month cannabis use and binge drinking compared to those who did not. Compared to those aged 50 to 64, those aged ≥65 were at lower risk for reporting both cannabis use and binge drinking (aPR = 0.36, 95% CI: 0.28–0.45). Compared to men, women were at lower risk (aPR = 0.45, 95% CI: 0.38–0.52). Compared to non-Hispanic white individuals, Hispanic individuals were at lower risk of both cannabis use and binge drinking (aPR = 0.61, 95% CI: 0.39–0.94), while non-Hispanic Black individuals were at higher risk (aPR = 1.28, 95% CI: 1.05–1.56). With regard to chronic conditions, those with a heart condition (aPR = 0.77, 95% CI: 0.60–0.98) and those with diabetes (aPR = 0.59, 95% CI: 0.46–0.76) were at lower risk. In addition, past-month tobacco use was associated with increased risk (aPR = 3.47, 95% CI: 2.98–4.04) as was past-year mental health treatment (aPR = 1.74, 95% CI: 1.39–2.16).

## Discussion

This study provides recent estimates of trends in prevalence of both past-month binge drinking and cannabis use among middle-aged and older adults in the US. We estimated that the prevalence of cannabis use among older adults has continued to increase based on previous estimates (Han & Palamar, 2020), with a 64.1% increase in past-month cannabis use between 2015 and 2019. Meanwhile, the prevalence of past-month binge drinking in this age group did not significantly change after previous increases over the past decade (Han, Moore, et al., 2019; Han, Moore, et al., 2017). However, there has been a 26.3% relative increase in the reported use of both cannabis and binge drinking in this population. This increase in binge drinking and cannabis use may partially be explained by the sharp decrease in perceptions of risk related to cannabis use among older adults, especially among those who report binge drinking (Han, Funk-White, Ko, Al-Rousan, & Palamar, 2021).

Older adults have a unique set of health considerations that are associated with higher morbidity and mortality compared to younger adults, including higher prevalence of chronic disease, prescription medication use, risk of falls, and geriatric conditions such as cognitive impairment and delirium (Cigolle, Langa, Kabeto, Tian, & Blaum, 2007). Among younger adults, simultaneous use of both alcohol and cannabis has been associated with higher impairment effects, higher consumption of both substances, impulsive behavior (i.e., drunk driving), higher rates of mental health diagnoses (including substance use disorders), and social consequences (i.e., personal, work-related, legal) (Subbaraman & Kerr, 2015; Yurasek et al., 2017). When examining cannabis use on its own, older adults who use cannabis have a higher risk of falls (Workman, Fietsam, Sosnoff, & Rudroff, 2021), ED visits related to injury (Choi, Marti, DiNitto, & Choi, 2018), longer hospitalizations when admitted for trauma (Lank & Crandall, 2014), and self-reported worse cognitive function (Benitez, Lauzon, Nietert, McRae-Clark, & Sherman, 2020). Similarly, excess alcohol use on its own has been shown extensively to be associated with negative health consequences for older adults, including cardiovascular disease, liver disease, cancer, falls and other injuries, and impaired physical and cognitive function (Han & Moore, 2018; Moore, Endo, & Carter, 2003; Ng Fat, Bell, & Britton, 2020; Ortolá et al., 2022). Prior studies have demonstrated an increased risk of cannabis use among younger people who engage in binge drinking, as well as negative health consequences related to co-use of these substances (DiNitto & Choi, 2011; Vijapur et al., 2021). Currently, the health outcomes of concurrent use among older adults are not clearly understood, and the rise in prevalence of both current binge drinking and cannabis use signals a need for urgency in addressing this knowledge gap.

Despite the lack of studies focused on older adults, we hypothesize that engaging in both excess alcohol and cannabis use is likely to increase the risk for medical and psychiatric comorbidities (Kuerbis, 2020). While our study did not show a significant increase in the prevalence of both binge drinking and cannabis use among older adults with selected chronic diseases, the use of both substances can place this population at high risk for not only exacerbation of health conditions, but complicating their management (Han, Ko, & Palamar, 2019). We also did find that receiving any mental health treatment in the past year was associated with reporting both binge drinking and cannabis use in this population. The intersection of multimorbidity and psychoactive substance use is complex, and the use of multiple substances can easily lead to harm among older adults.

Additionally, the association of current tobacco use in addition to both binge drinking and cannabis use adds increasing concern for health risks in this population. The use of multiple psychoactive substances with tobacco use can have serious health consequences and risk of death (Subbaraman & Kerr, 2015; Xu et al., 2007). This highlights the need for clinicians to screen older adults for the use of multiple psychoactive substances and to communicate the risks for interactions with existing chronic diseases and prescribed medications (Han & Moore, 2018). However, rates of screening and discussion around psychoactive substance use remain low for older adults (Han & Moore, 2018; Kuerbis, 2020; Mauro, Askari, & Han, 2021). Given the increased risk for harm, all older adults should be screened and counseled regarding their use of alcohol, drugs, and tobacco while also providing evidence-based interventions for older adults who meet the criteria for substance use disorder.

While our results show an increasing trend in reporting both current cannabis use and binge drinking among older adults who identify as non-Hispanic white, we also found that compared to non-Hispanic white individuals, non-Hispanic Black individuals aged ≥50 were more likely to report both. Studies on concurrent use of alcohol and cannabis among older adults by race and ethnicity are lacking, but limited data suggest that non-Hispanic Black adults report higher prevalence of concurrent use, and that Hispanic adults report lower levels of concurrent use than non-Hispanic white adults (Banks, Rowe, Mpofu, & Zapolski, 2017; Subbaraman & Kerr, 2015). For adolescents, research suggests that rates of concurrent alcohol and cannabis use have increased disproportionately among non-Hispanic Black individuals (Lanza, Vasilenko, Dziak, & Butera, 2015), while for adults, Midanik, Tam, and Weisner found that identifying as Black was associated with simultaneous use of cannabis and alcohol, but not concurrent use (Midanik et al., 2007). As cannabis use continues to increase sharply among older adults across all races and ethnicities (Han & Palamar, 2020), it will be increasingly important to recognize differences in the use of both excess alcohol and cannabis to inform targeted interventions.

This study has several limitations. The cross-sectional nature of the study hinders establishing any temporal relationships. Furthermore, since this study focuses on the past-month use of both binge drinking and cannabis use only, we cannot ascertain whether respondents used these substances at the same time or separately. NSDUH relies on self-report and is subject to limited recall and social desirability bias. The latter may be particularly true for drug and alcohol use in which respondents may not report their psychoactive substance use due to perceived stigma, although the survey attempts to limit this via the use of ACASI approach to limit potential social desirability bias. NSDUH also samples only the noninstitutionalized U.S. population and therefore does not include adults living in long-term care settings or who may be experiencing homelessness, which limits generalizability to these populations. Finally, because of methodological and data collection changes due to the COVID-19 pandemic, we were unable to include the 2020 NSDUH data for this study.

## Conclusion

The estimated prevalence of both past-month cannabis use and binge drinking has continued to increase with the overall increase in current cannabis use among adults aged 50 and older in the US. Screening for and discussions around psychoactive substance use and excessive alcohol use are increasingly important for middle-aged and older adults living with chronic diseases.

## Figures and Tables

**Table 1:** Trends in prevalence estimates for self-reported both past-month cannabis use and binge drinking by sociodemographic, chronic disease, and substance use characteristics for persons 50 years and older, United States 2015–2019

All adults 50 and older	2015	2016	2017	2018	2019	% absolute change, 2015–2019	% relative change, 2015–2019	*p* value
Prevalence of past-month cannabis use	3.9	4.1	4.8	4.9	6.4	2.5	64.1	<0.001
Prevalence of past-month binge drinking	18.1	17.1	17.6	17.7	17.3	−0.8	−4.4	0.46
Prevalence of past-month binge drinking and cannabis use	1.9	1.6	2.0	2.0	2.4	0.5	26.3	0.03
**Prevalence of past-month binge drinking and cannabis use**								
Age								
50–64	3.1	2.3	2.9	3.0	3.5	0.4	12.9	0.14
≥65	0.2	0.7	0.8	0.8	1.1	0.9	450.0	<0.001
**Sex**								
Male	2.7	2.6	2.9	3.0	3.5	0.8	29.6	0.12
Female	1.1	0.8	1.2	1.2	1.5	0.4	36.4	0.06
**Race/ethnicity**								
Non-Hispanic White	1.8	1.7	2.1	2.2	2.4	0.6	33.3	0.03
Other	2.0	1.3	1.5	1.7	2.5	0.5	25.0	0.33
**Annual total family income**								
<$20,000	2.2	1.9	3.1	2.7	4.1	1.9	86.4	0.01
$20-$49,999	1.7	1.5	1.8	1.7	2.1	0.4	23.5	0.33
$50,000-$74,999	1.9	1.5	1.4	2.3	2.5	0.6	31.6	0.18
≥$75,000	1.8	1.6	1.9	1.9	2.1	0.3	16.7	0.46
**Chronic disease**								
Asthma	2.2	1.2	1.3	1.8	2.3	0.1	4.5	0.66
Cancer	1.0	0.7	1.0	1.2	1.4	0.4	40.0	0.34
Cardiovascular disease	0.8	1.3	1.6	1.5	1.7	0.9	112.5	0.11
Chronic obstructive pulmonary disease	1.8	1.4	2.7	3.0	2.5	0.7	38.9	0.09
Diabetes	1.0	0.7	1.0	1.7	1.2	0.2	20.0	0.20
Hypertension	1.6	1.5	1.0	1.9	2.0	0.4	25.0	0.23
2 or more chronic diseases ^a^	1.3	1.3	0.9	1.6	1.9	0.6	46.2	0.14
**Substance use (past-month)**								
Tobacco use	5.6	4.4	5.7	5.9	7.4	1.8	32.1	0.06
**Health care utilization (past-year)**								
All-cause emergency department use	2.0	1.5	2.3	2.0	2.9	0.9	45.0	0.03
Received any mental health treatment	2.5	2.9	3.8	3.4	3.9	1.4	56.0	0.07

a Includes asthma, cancer, cardiovascular disease, chronic obstructive pulmonary disease, cirrhosis, diabetes, hepatitis B or C, HIV/AIDS, hypertension, and kidney disease.

**Table 2:** Correlates of reporting past-month cannabis use and binge drinking among adults age ≥50, United States 2015–2019

Characteristic	Full sample (n = 44,007), weighted % (95% CI)	No past-month cannabis use and binge-drinking (n = 43,073), weighted % (95% CI)	Past-month cannabis use and binge-drinking (n = 934), weighted % (95% CI)	χ^2^ *p* value	aPR^a^ (95% CI)
**Age**					
50–64	55.6 (55, 56.2)	55.1 (54.4, 55.7)	83.2 (80.0, 86.0)	<0.001	Ref
≥65	44.4 (43.8, 45)	44.9 (44.3, 45.6)	16.8 (14.0, 20.0)		0.36 (0.28, 0.45)
**Sex**					
Male	46.8 (46.1, 47.4)	46.3 (45.7, 46.9)	69.3 (65.8, 72.5)	<0.001	Ref
Female	53.2 (52.6, 53.9)	53.7 (53.1, 54.3)	30.7 (27.5, 34.2)		0.45 (0.38, 0.52)
**Race/ethnicity**					
Non-Hispanic White	72.4 (71.6, 73.1)	72.3 (71.6, 73.1)	75.2 (72.0, 78.1)	<0.001	Ref
Non-Hispanic African American	10.5 (10.0, 10.9)	10.4 (10.0, 10.8)	15.0 (12.7, 17.6)		1.28 (1.05, 1.56)
Hispanic	10.7 (10.2, 11.3)	10.8 (10.3, 11.4)	6.0 (3.9, 9.1)		0.61 (0.39, 0.94)
Other	6.4 (6.1, 6.8)	6.5 (6.14, 6.86)	3.8 (2.6, 5.4)		0.64 (0.44, 0.91)
**Annual total family income**					
<$20,000	15.4 (14.9, 15.9)	15.3 (14.8, 15.8)	21.5 (18.5, 24.8)	0.002	Ref
$20-$49,999	29.9 (29.3, 30.5)	30.0 (29.4, 30.6)	26.6 (22.7, 30.8)		0.86 (0.68, 1.09)
$50,000-$74,999	16.3 (15.9, 16.7)	16.3 (15.9, 16.8)	15.7 (12.6, 19.3)		0.89 (0.67, 1.18)
>$75,000	38.4 (37.6, 39.2)	38.4 (37.6, 39.3)	36.3 (32.5, 40.2)		0.81 (0.65, 1.02)
**Chronic disease**					
Asthma	7.9 (7.6, 8.3)	8.0 (7.6, 8.3)	7.0 (5.2, 9.5)	0.41	0.95 (0.65, 1.39)
Cancer	11.8 (11.4, 12.1)	11.9 (11.5, 12.2)	6.3 (4.7, 8.5)	<0.001	0.75 (0.54, 1.04)
Cardiovascular disease	18.7 (18.3, 19.2)	18.9 (18.4, 19.4)	13.0 (10.6, 15.7)	<0.001	0.77 (0.60, 0.98)
Chronic Obstructive Pulmonary Disease	7.4 (7.1, 7.7)	7.4 (7.0, 7.7)	8.6 (6.6, 11.1)	0.24	1.00 (0.70, 1.42)
Diabetes	18.0 (17.5, 18.5)	18.1 (17.6, 18.7)	10.3 (8.3, 12.9)	<0.001	0.59 (0.46, 0.76)
Hypertension	33.0 (32.5, 33.5)	33.1 (32.6, 33.6)	26.6 (23.0, 30.5)	0.002	0.97 (0.77, 1.23)
2 or more chronic diseases^b^	25.9 (25.5, 26.4)	26.1 (25.6, 26.5)	18.4 (15.7, 21.5)	<0.001	1.07 (0.76, 1.51)
**Substance use (past-month)**					
Tobacco use	18.5 (18.0, 19.0)	17.8 (17.3, 18.3)	54.1 (50.2, 57.8)	<0.001	3.47 (2.98, 4.04)
**Health care utilization (past-year)**					
All-cause emergency department use	26.0 (25.4, 26.6)	25.9 (25.4, 26.5)	28.1 (24.1, 32.6)	0.30	1.05 (0.84, 1.31)
Received any mental health treatment	14.0 (13.6, 14.4)	13.8 (13.5, 14.2)	23.2 (19.6, 27.3)	<0.001	1.74 (1.39, 2.16)

aPR = adjusted prevalence ratio; CI: confidence interval.

a Adjusted for all listed covariates including survey year.

b Includes asthma, cancer, cardiovascular disease, chronic obstructive pulmonary disease, cirrhosis, diabetes, hepatitis B or C, HIV/AIDS, hypertension, and kidney disease.
